# Breast cancer metastases to the thyroid gland – an uncommon sentinel for diffuse metastatic disease: a case report and review of the literature

**DOI:** 10.1186/s13256-017-1441-x

**Published:** 2017-09-22

**Authors:** Agata M. Plonczak, Aimee N. DiMarco, Roberto Dina, Dorothy J. Gujral, Fausto F. Palazzo

**Affiliations:** 1 Department of Thyroid & Endocrine Surgery, Hammersmith Hospital, Imperial College Hospitals NHS Trust, London, W12 0HS UK; 2 Department of Histopathology, Hammersmith Hospital, Imperial College Hospitals NHS Trust, London, W12 0HS UK; 3 Department of Oncology, Charing Cross Hospital, Imperial College Hospitals NHS Trust, London, W6 8RF UK

**Keywords:** Thyroid disorders, Breast cancer, Clinical oncology, Endocrine surgery

## Abstract

**Background:**

Metastases to the thyroid are rare. The most common primary cancer to metastasize to the thyroid is renal cell carcinoma, followed by malignancies of the gastrointestinal tract, lungs, and skin, with breast cancer metastases to the thyroid being rare. Overall, the outcomes in malignancies that have metastasized to the thyroid are poor. There are no prospective studies addressing the role of surgery in metastatic disease of the thyroid. Isolated thyroidectomy has been proposed as a local disease control option to palliate and prevent the potential morbidity of tumor extension related to the airway. Here, we present a case of a patient with breast cancer metastases to the thyroid gland and discuss the role of thyroidectomy in the context of the current literature.

**Case presentation:**

A 62-year-old Afro-Caribbean woman was diagnosed as having bilateral breast carcinoma in 2004, for which she underwent bilateral mastectomy. The pathology revealed multifocal disease on the right, T2N0(0/20)M0 grade 1 and 2 invasive ductal carcinoma, and on the left side, T3N1(2/18)M0 grade 1 invasive ductal carcinoma. Surgery was followed by adjuvant chemotherapy and regional radiotherapy. The disease was under control on hormonal therapy until 2016, when she developed cervical lymphadenopathy. The fine-needle aspiration cytology of the thyroid was reported as papillary thyroid cancer; and the fine-needle biopsy of the left lateral nodal disease was more suggestive of breast malignancy. She underwent a total thyroidectomy and a clearance of the central compartment lymph nodes and a biopsy of the lateral nodal disease. The histopathological analysis was consistent with metastatic breast cancer in the thyroid and lymph nodes with no evidence of a primary thyroid malignancy.

**Conclusions:**

A past history of a malignancy elsewhere should raise the index of suspicion of metastatic disease in patients presenting with thyroid lumps with or without cervical lymphadenopathy. Detection of metastases to the thyroid generally indicates poor prognosis, obviating the need of surgery in an already compromised patient. An empirical thyroidectomy should be considered in select patients for local disease control.

## Background

Breast cancer is the most commonly diagnosed cancer among women [1]. The common sites for metastatic spread are bone, lungs, and liver [2]. Metastases to the thyroid gland from a non-thyroid primary are uncommon and are mostly from the kidney, followed by gastrointestinal tract, lungs, skin, and rarely breast [3–7]. It is usually associated with a poor prognosis. There are no prospective studies addressing the role of surgery in metastatic disease of the thyroid. Isolated thyroidectomy has been proposed as a local disease control option to palliate and prevent the potential morbidity of tumor extension related to the airway. Here, we present a rare case of a patient with breast cancer metastases to the thyroid gland, and review the evidence for the role of thyroidectomy in the context of the current literature.

## Case presentation

**Fig. 1:**
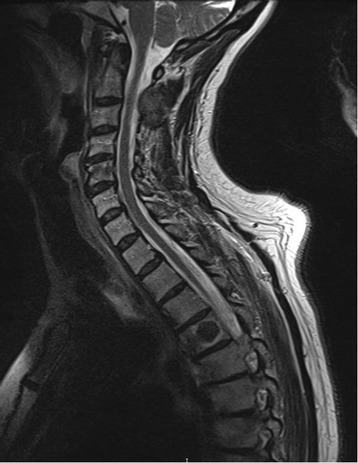
T2-weighted sagittal magnetic resonance image demonstrating the deposits in C5 and T4. They appeared confined to the vertebral body with no evidence of vertebral body collapse

**Fig. 2:**
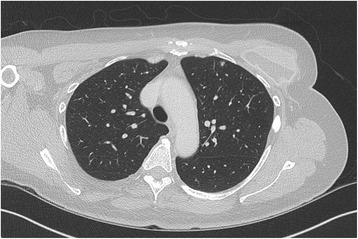
Computed tomography of the thorax demonstrating a small (5 mm in diameter) subpleural nodule within the anterior left upper lobe, which remained unchanged since the previous scan

**Fig. 3:**
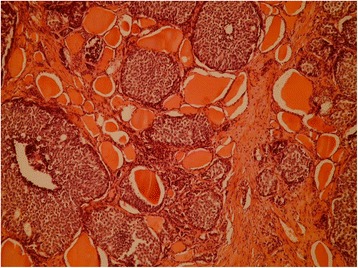
Hematoxylin and eosin stain at × 100 magnification demonstrating solid nests of atypical epithelial cells among normal colloid-filled thyroid follicles

**Fig. 4:**
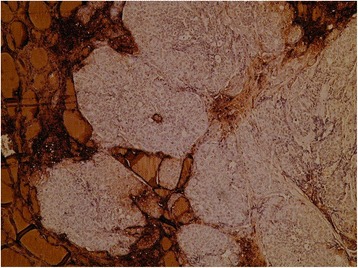
Immunoperoxidase for thyroglobulin showing the solid nests, which are negative while the follicles are positive, including a small trapped microfollicle within the larger nest of metastatic cells. Thyroid transcription factor 1 and calcitonin were equally negative; however, cytokeratin 7 was focally positive and synaptophysin was expressed by the majority of cells. This raises the possibility of a carcinoma with neuroendocrine features

A 62-year-old Afro-Caribbean woman was diagnosed as having bilateral carcinoma of the breast in 2004. Her past medical history included hypertension, controlled by amlodipine and losartan, in addition to diabetes on treatment with metformin. She underwent bilateral mastectomy and axillary node clearance with immediate implant-based reconstruction. The pathology revealed multifocal disease on the right, T2N0(0/20)M0 grade 1 and 2 invasive ductal carcinoma (IDC), and on the left side, T3N1(2/18)M0 grade 1 IDC. The disease was estrogen receptor (ER)-positive, weak progesterone receptor (PR)-positive, and human epidermal growth factor receptor 2 (HER2)-negative. Surgery was followed by adjuvant chemotherapy, consisting of the 5-fluorouracil, epirubicin, and cyclophosphamide (FEC) regimen and regional radiotherapy. Hormonal therapy initially consisted of 20 mg daily of tamoxifen. After 3 years this was switched to an aromatase inhibitor (anastrozole 1 mg daily) until 2009 when she completed 5 years of adjuvant endocrine therapy. She then subsequently relapsed with metastatic disease with lung nodules in 2008 and bone metastases were noted on a bone scan 4 years later. She was commenced on 25 mg once a day of exemestane and 4 mg intravenously administered monthly injections of zoledronic acid in early 2014. Due to disease progression, capecitabine 1250 mg/m^2^ (based on total body surface area) twice daily was commenced until after six cycles when it was discontinued due to capecitabine-related toxicity and she was started on 2.5 mg once a day of letrozole and 150 mg once a day of ibandronic acid. In February 2016 she presented with neck swelling with intermittent neck discomfort without airway pressure symptoms. On clinical examination she was found to have cervical lymphadenopathy. Laboratory findings revealed a white cell count of 5.2 × 10^9^/L, hemoglobin of 115 g/L, and normal liver and renal function with an estimated glomerular filtration rate of 67 ml/minute/1.73 m^2^. The neck swelling was investigated with an ultrasound and confirmed both lateral cervical nodal disease in levels II to IV and a goiter with left-sided dominance. The fine-needle aspiration cytology (FNAC) of her thyroid was reported as in keeping with a papillary thyroid cancer; however, the cytology of the left lateral nodal disease was described as more suggestive of a breast malignancy. She had no personal or familial risk factors for thyroid malignancy. Staging investigations including magnetic resonance imaging (MRI) of her spine demonstrated stable deposits involving C2, C5, T4, and L1 without neural compromise (Fig. 1) and computed tomography (CT) of her thorax demonstrated no change in the lung nodules (Fig. 2). Since the diagnosis was not clear, following a multidisciplinary team discussion the decision was made to proceed with a total thyroidectomy and a clearance of the central compartment lymph nodes coupled with an excision biopsy of the laterocervical lymph nodes. Histopathological analysis of the specimen demonstrated an ill-circumscribed white tumor at the posterior margin of the left lobe measuring 1.2 × 0.9 × 1.5 cm. On immunocytochemistry the tumor cells were positive for carcinoembryonic antigen (CEA), synaptophysin, GATA3, and ER (5/8), focally positive for cytokeratin (CK) 7 and gross cystic disease fluid protein 15 (GCDFP-15), and negative for thyroid transcription factor 1 (TTF-1), calcitonin, thyroglobulin, CK20, PR, and HER2. The overall appearances were consistent with metastatic breast cancer (Figs. 3 and 4) with no evidence of a primary thyroid malignancy. The level IV and level VI lymph nodes contained metastatic breast cancer. She was discharged on daily 125 mcg of levothyroxine. The chemotherapy was switched to 500 mg intramuscular monthly injections of fulvestrant and she continues to take the ibandronic acid. Currently, 14 months following the thyroidectomy, she remains clinically stable. She developed local recurrences in the level II to IV lymph nodes in her neck and a recent MRI of her spine showed stable spinal metastatic disease.

## Discussion

Metastatic deposits have a predilection for highly vascularized organs but despite one of the highest blood supplies per weight of tissue (4 to 6 mL/minute/g) [8] the thyroid is rarely the site for metastatic deposits. It is difficult to establish the true rate of metastases from breast cancer to the thyroid gland with a quoted range of prevalence from 3% of all thyroid metastases [4] to 34% (Table 1) [3, 4, 6, 7, 9–27]. Metastases to the thyroid gland represent an indication for surgery in under 1 in a 1000 thyroidectomies [24, 28] of which almost half are from a renal cell carcinoma primary [7, 29]. Other primary tumors that have been documented to metastasize to the thyroid include colorectal, lung, and malignant melanoma [3–7] and gastrointestinal tract tumors [10].

**Table 1:** Clinical studies (case reports and case series) of breast metastases to the thyroid gland published so far

Author	Study years	Number of patients	Percentage of thyroid metastases from breast
Harcourt-Webster [9]	–	2	18%
Lam and Lo [10]	–	7	9%
Mayo and Schlicke [11]	–	2	11%
Elliott and Frantz [12]	1947–1958	4	29%
Wychulis *et al.* [13]	1907–1962	4	29%
Pillay *et al.* [14]	1974–1976	1	10%
Lin *et al.* [15]	1977–1995	1	7%
Chacho *et al.* [16]	1978–1985	1	13%
Rosen *et al.* [17]	1978–1993	1	9%
Hegerova *et al.* [7]	1980–2010	11	11%
De Ridder *et al.* [18]	1982–2002	1	17%
Russell *et al.* [19]	1983–2013	2	12%
Cichon *et al.* [20]	1984–2003	1	6%
Nakhjavani *et al.* [21]	1985–1994	7	16%
Wood *et al.* [22]	1985–2002	1	7%
HooKim *et al.* [6]	1986–2013	3	11%
Saito *et al.* [23]	1987–2008	3	34%
Papi *et al.* [24]	1993–2003	5	14%
Moghaddam *et al.* [3]	1993–2013	1	10%
Calzolari *et al.* [25]	1995–2005	1	4%
Kim *et al.* [26]	1997–2004	5	23%
Surov *et al.* [4]	1997–2013	1	3%
Choi *et al.* [27]	2001–2013	7	15%

Breast cancer is the most common malignant tumor among women [1]; while being uncommon, thyroid cancers are the most common endocrine malignancies and the incidence is rising [30]. It has been suggested that possibly due to some common risk factors (genetic, lifestyle, diet habits, hormonal, menstrual, and reproductive factors), individuals with breast cancer are more likely to develop primary thyroid cancer [31, 32]. Therefore, an individual presenting with both thyroid and breast malignancy is more likely to have primary cancer of thyroid and breast, rather than breast metastases to the thyroid.

Up to 80% of thyroid metastases are metachronous [29] with mean intervals from as little as 2.3 years in head and neck cancer [7, 21] to as long as 21 years in the case of foregut neuroendocrine tumors [33]. Other metachronous tumors present varying levels of delay with a mean of 9.4 years in renal cell carcinoma primaries [34] and 48.2 months [29] in breast primary malignancies. Longer delays in metachronous tumors probably reflect a less aggressive biology and in fact the rarer synchronous metastases to the thyroid are associated with a much poorer prognosis with a mean 5-year survival rate of 7.9% [35].

Most reports of metastases to the thyroid are solitary with Surov and colleagues [4] reporting that thyroid metastases were solitary in 76% of patients in their study. However, Hegerova *et al.* [7] reported that 79% of their patients had evidence of other metastases at the time of diagnosis of thyroid metastases, which may suggest that the extent of investigations plays a part in determining the identification of other disease.

FNAC is the investigation of choice in the work-up of thyroid nodules. It has been shown to achieve an accuracy of over 90% in the diagnosis of secondary tumors of the thyroid [36]. Unfortunately, as in the case presented, metastatic ductal breast carcinoma involving the thyroid may morphologically mimic primary thyroid malignancy on fine-needle aspiration (FNA) and secondary malignancies of the thyroid may be misdiagnosed.

Outcomes in metastatic thyroid disease tend to be poor since it is a reflection of the aggression and advanced stage of the primary disease [5, 15]. A Mayo Clinic series demonstrated that the mean survival post diagnosis of metastases to the thyroid is 3 years and 6 years from the original diagnosis of a primary malignancy [7].

There are no prospective studies addressing the role of surgery in metastatic disease of the thyroid. Our patient with breast metastasis to the thyroid and coexisting lung and bone metastatic deposits, was managed with a total thyroidectomy with a good outcome. Isolated thyroidectomy has been proposed in previous studies [20, 37] as a local disease control option to palliate and prevent the potential morbidity of tumor extension related to the airway [37]. It has been also suggested that this may be beneficial for a selected group of patients with clinically significant and relatively isolated metastatic disease of the thyroid especially from a renal primary [25]; however, in the absence of prospective trials this is at best speculative.

## Conclusions

A past history of a malignancy elsewhere should raise the index of suspicion of metastatic disease in patients presenting with thyroid lumps with or without cervical lymphadenopathy. Detection of metastases to the thyroid generally indicates poor prognosis, obviating the need of surgery in an already compromised patient. An empirical thyroidectomy should be considered in select patients for local disease control.
