# Correction to: Breast cancer metastases to the thyroid gland - An uncommon sentinel for diffuse metastatic disease: A case report and literature review

**DOI:** 10.1186/s13256-017-1478-x

**Published:** 2017-10-06

**Authors:** Agata M. Plonczak, Aimee N. DiMarco, Roberto Dina, Dorothy M. Gujral, Fausto F. Palazzo

**Affiliations:** 1 Department of Thyroid & Endocrine Surgery, Hammersmith Hospital, Imperial College Hospitals NHS Trust, London, W12 0HS UK; 2 Department of Histopathology, Hammersmith Hospital, Imperial College Hospitals NHS Trust, London, W12 0HS UK; 3 Department of Oncology, Charing Cross Hospital, Imperial College Hospitals NHS Trust, London, W6 8RF UK

## Correction

In the publication of this article [1], there is an error in an authors name.

The error: ‘Dorothy J Gujral’ Should instead read: ‘Dorothy M Gujral’

This has now been updated in the original article [1].
